# A Genome-Wide Association Study of Field Resistance to *Magnaporthe Oryzae* in Rice

**DOI:** 10.1186/s12284-016-0116-3

**Published:** 2016-08-30

**Authors:** Dan Zhu, Houxiang Kang, Zhiqiang Li, Minghao Liu, Xiaoli Zhu, Yue Wang, Dan Wang, Zhilong Wang, Wende Liu, Guo-Liang Wang

**Affiliations:** 1 State Key Laboratory for Biology of Plant Diseases and Insect Pests, Institute of Plant Protection, Chinese Academy of Agricultural Sciences, Beijing, 100193 People’s Republic of China; 2 Hunan Provincial Key Laboratory of Crop Germplasm Innovation and Utilization and College of Agronomy, Hunan Agricultural University, Changsha, Hunan 410128 People’s Republic of China; 3 Department of Plant Pathology, Ohio State University, Columbus, OH 43210 USA

**Keywords:** Rice, *Magnaporthe oryzae*, GWAS, Field blast resistance, LAFBR

## Abstract

**Background:**

Breeding of rice cultivars with long-lasting resistance to the rice blast fungus *Magnaporthe oryzae* is difficult, and identification of new resistance genes is essential. Most of the loci associated with blast resistance against *M. oryzae* in rice have been identified in controlled environments and with single isolates, and such loci may confer resistance to only a small faction of the *M. oryzae* strains. In the field, however, rice is commonly attacked by multiple strains. Research is therefore needed to identify loci that confer resistance in the field, i.e., “field blast resistance”. To identify loci associated with field blast resistance (LAFBRs), we conducted a genome-wide association study (GWAS) using the rice diversity panel 1 (RDP1) cultivars. These cultivars were evaluated in the field in three major rice production areas of China.

**Results:**

GWAS identified 16 LAFBRs. Among them, 13 are novel and the other three are co-localized with known blast resistance regions. Seventy-four candidate genes are identified in the 16 LAFBR regions, which encode receptor-like protein kinases, transcription factors, and other defense-related proteins. Using the rice transcriptome data, compared with the rice-rice blast compatible interaction, we identified seven candidate genes that are significantly up-regulated and five genes that are significantly down-regulated in the incompatible interaction among the candidate genes.

**Conclusions:**

We identified 16 LAFBRs involved in field resistance to *M. oryzae* and 20 cultivars that exhibit high levels of resistance in both the field and growth chamber. The resistant cultivars and the SNP markers identified in this study should be useful for marker-assisted selection of new rice cultivars that confer high levels of resistance against *M. oryzae* field populations.

**Electronic supplementary material:**

The online version of this article (doi:10.1186/s12284-016-0116-3) contains supplementary material, which is available to authorized users.

## Background

Rice (*Oryzae sativa* L.) is an important food crop that feeds more than half of the world’s population (Khush 2005). Rice blast, caused by the fungal pathogen *Magnaporthe oryzae*, is a destructive disease of rice that reduces yields from 10 to 30 % annually (Skamnioti and Gurr 2009). The most effective and economical way to control the disease is via resistant cultivars (Hulbert et al., 2003). To date, more than 100 blast resistance (R) genes and about 500 quantitative trait loci (QTLs) have been identified (Ashkani et al., 2015), and 25 of them have been cloned (Wu et al., 2015; Zheng et al. 2016). However, rice cultivars often lose their resistance to *M. oryzae* within 3–5 years because of the high variability of the fungus in the field (Oliveira-Garcia and Valent 2015; Devi et al. 2015). In major production areas in China, for example, 174 resistant rice cultivars (disease index <4 on a scale from 0 to 9) released from 2004 to 2008 lost their blast resistance (Feng et al. 2014). It is therefore necessary to identify new rice blast R genes that will be effective against *M. oryzae* field populations for extended periods.

The classic genetic linkage mapping strategy using bi-parental crosses has been widely used to identify R genes and QTLs. Because this strategy requires the construction of a mapping population and genotyping, however, it is labor intensive and time consuming. The genome-wide association study (GWAS) method based on the high-density SNP markers has recently been established in plants such as maize (Yu et al. 2006), rice (Huang et al. 2010 and Zhao et al. 2011), and soybean (Hwang et al. 2014). Compared with the traditional bi-parental mapping strategy, the genetic background of the population for GWAS is much more diverse, which can be used for mapping of rare alleles of agronomic traits. Recently, in rice, dozens of new genes/QTLs associated with different phenotypes have been identified with GWAS because the method efficiently dissects the genetic structure of complicated phenotypes (Zhao et al. 2011; Spindel et al. 2016). The rice diversity panel 1 (RDP1), which consists of over 400 *O. sativa* cultivars collected from 82 countries, is publically available and contains substantial genetic and phenotypic diversity (Eizenga et al. 2014; Ali et al. 2010; Eizenga et al. 2013). Importantly, a high-density SNP map for the RDP1 has been generated and is publically available. Researchers have used the RDP1 to identify many genes/QTLs associated with important agronomic traits in rice (Zhao et al. 2011; Norton et al. 2014; Copenhaver et al. 2011). Using the same RDP1 and growth chamber assays, Kang et al. (2016) recently identified 97 loci associated with blast resistance (LABRs) against five *M. oryzae* isolates.

In this study, we evaluated the resistance of the RDP1 cultivars in rice blast nurseries (field sites containing highly diverse *M. oryzae* populations) in three representative rice production regions of China. Association mapping showed that 16 loci associated with field blast resistance (LAFBRs) are significantly linked to rice blast field resistance. The resistant cultivars and the LAFBRs identified in this study will be useful for the breeding of blast resistance in rice.

## Results

### Evaluation of the Field Blast Resistance of the RDP1 Cultivars

**Fig. 1:**
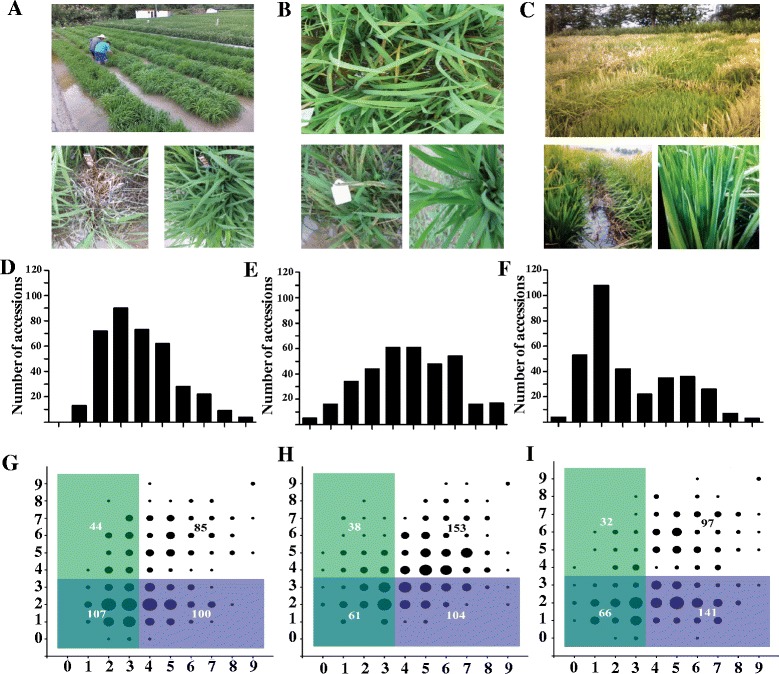
Field blast evaluation of the RDP1 cultivars and disease resistance scores of the cultivars in the three locations. **a–c** Seedlings of RDP1 in Shanghang (Fujian province), Taojiang (Hunan province) and Wuchang (Heilongjiang province) blast nurseries, respectively**. d–f** Distribution of blast disease resistance scores of RDP1 in Shanghang, Taojiang and Wuchang respectively. *X axis* represents the disease scales, *Y-axis* represents the number of cultivars. **g** Pair-wise comparison of disease resistance between Shanghang (*X-axis*) and Wuchang (*Y-axis*). **h** Pair-wise comparison of disease resistance between Taojiang (*X-axis*) and Shanghang (*Y-axis*). **i** Pair-wise comparison of disease resistance between Taojiang (*X-axis*) and Wuchang (*Y-axis*). The area of black circles represents the accession numbers. The overlap region with double colors represents the resistant cultivars (0–3) at both locations and the single color region represents the resistant accessions (0–3) at one location but susceptible (4–9) at the other location

A total of 373, 356, and 336 rice cultivars in the RDP1 were grown in the blast nurseries of the three Chinese rice production areas: Shanghang (in Southeast China, Fujian Province), Wuchang (in Northeast China, Heilongjiang Province), and Taojiang (in Central China, Hunan Province) (Fig. 1a–c; Additional file 1: Table S1). Consistent with previous results obtained under growth chamber conditions (Kang et al. 2016), a large range of resistant phenotypes in the RDP1 were detected in the three nurseries. At Wuchang, 61.6 % (207 of 336) of the cultivars were resistant (with a score of 0 to 3 on a disease severity scale from 0 to 9) (Fig. 1d), compared to 46.9 % (175 of 373) at Shanghang and 27.8 % (99 of 356) at Taojiang (Fig. 1e, f). These results indicated that the blast disease pressure was highest at the Taojiang site.

To understand the differences in resistance among the cultivars at the three sites, we performed pair-wise comparisons of cultivars’ disease reactions. The analysis showed that 70.9 % (107 of 151) of the resistant cultivars at Shanghang were also resistant at Wuchang. In contrast, only 51.7 % (107 of 207) of the resistant cultivars at Wuchang were resistant at Shanghang (Fig. 1g). Of the resistant cultivars at Taojiang, 61.6 % (61 of 99) were also resistant at Shanghang. In contrast, only 37.0 % (61 of 165) of the resistant cultivars at Shanghang were also resistant at Taojiang (Fig. 1h). A high percentage of resistant cultivars (67.3 %, 66 out of 98) at Taojiang were also resistant at Wuchang but only 31.9 % (66 of 207) of the resistant cultivars at Wuchang were resistant at Taojiang (Fig. 1i). These results suggest that the RDP1 cultivars that are highly resistant to the *M. oryzae* population at Taojiang have broad-spectrum resistance. Further analysis revealed that 40 cultivars were resistant to the *M. oryzae* populations at all three locations (Additional file 2: Table S2). Of these 40 cultivars, 20 were also resistant to the five *M. oryzae* isolates collected from five countries (Kang et al. 2016). Information on these 20 cultivars is provided in Table 1.

**Table 1:** Information concerning the 20 cultivars of rice that were highly resistant to rice blast disease under both field and growth chamber conditions

Accession or name	GSOR ID	IRGC ID	NSFTV. ID	Sub-population	Origin
Dom-sufid	301042	117721	45	ARO	Iran
Geumobyeo	301052	117612	56	TEJ	South Korea
IRAT 177	301066	117761	73	TRJ	French Guiana
Jambu	301068	117764	75	TRJ	Indonesia
LAC 23	301091	117796	99	TRJ	Liberia
NSF-TV 116	301108	117824	116	TRJ	Pakistan
B6616A4-22-Bk-5-4	301158	117646	167	TRJ	United States
Zhenshan 2	301163	117944	172	IND	China
IRAT 13	301186	117760	195	TRJ	Cote D’Ivoire
WAB 502-13-4-1	301229	117932	239	TRJ	Cote D’Ivoire
SL 22-613	301284	117756	294	ADM	Sierra Leone
Llanero 501	301298	117805	308	TRJ	Venezuela
Manzano	301299	To be assigned	309	TRJ	Zaire
R 101	301300	117857	310	TRJ	Zaire
DNJ 140	301313	117718	323	AUS	Bangladesh
Berenj	301330	117656	340	ADM	Afghanistan
OS 6 (WC 10296)	301378	117830	395	TRJ	Zaire
Cocodrie	301379	117693	396	TRJ	United States
Saber	301411	To be assigned	630	TRJ	United States
C101A51	301420	To be assigned	626	IND	Colombia

*GSOR ID* Genetic Stocks-*Oryza* collection identification number, *IRGC ID* International Rice Germplasm Collection identification number

**Fig. 2:**
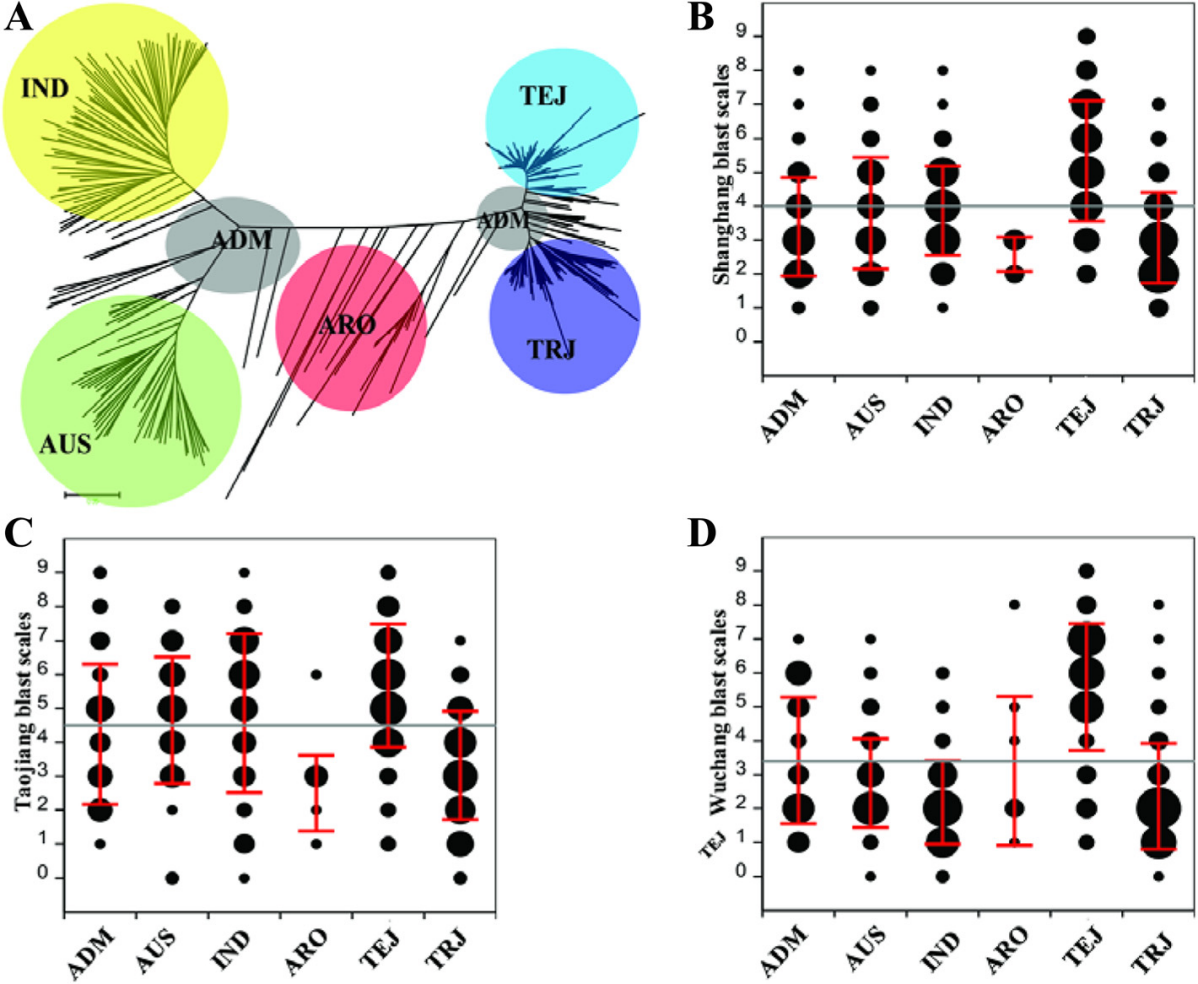
Grouping of the RDP1 cultivars and blast resistance of the different sub-populations. **a** Phylogenetic tree of RDP1. ADM = *admixture*; ARO = *aromatic*; AUS = *aus*; IND = *indica*; TEJ = *temperate japonica*; TRJ = *tropic japonica*. Distribution of blast resistance scores of RDP1 in the six sub-populations in Shanghang (**b**), Taojiang (**c**) and Wuchang (**d**), respectively. The area of black circles represent the accession numbers, the *red line* represents the standard deviation (SD) of the blast resistance scores in each sub-population, and the *gray line* represents the average level of the blast resistance scores of the RDP1 cultivars

Based on the evaluation of disease at the three field sites, we analyzed the differences in rice blast resistance among sub-populations of the RDP1. First, we constructed the phylogenetic tree of the RDP1 cultivars using 3835 high quality SNP markers selected from the 44-K SNP dataset (Zhao et al. 2011). The analysis showed that the RDP1 has five major sub-populations (*indica* [IND], *aus* [AUS], *tropical japonica* [TRJ], *temperate japonica* [TEJ], and *aromatic* [ARO]) and an additional sub-population (*admixture* [ADM]). The genetic distance between TRJ and TEJ is very small (Fig. 2a). When the blast scores were classified according to the sub-populations, we found two main features. First, the ratio of resistant to susceptible cultivars in AUS and IND sub-populations was close to 0.5 in Central and Southeastern China (Taojiang and Shanghang) but was greater than 0.5 in the two sub-populations in Northeastern China (Wuchang). Second, in all three areas, more than half of the cultivars (68.6, 51.3, and 82.4 % in Shanghang, Taojiang, and Wuchang, respectively) in the TRJ sub-population were resistant, and most of the cultivars (84.4, 89.4, and 83.9 % in Shanghang, Taojiang, and Wuchang, respectively) in the TEJ sub-population were susceptible (Fig. 2b–d). Although the genetic distance between TRJ and TEJ sub-populations is less than that between the other sub-populations (Fig. 2a), blast resistance is higher in TRJ than TEJ.

### Identification of LAFBRs

**Fig. 3:**
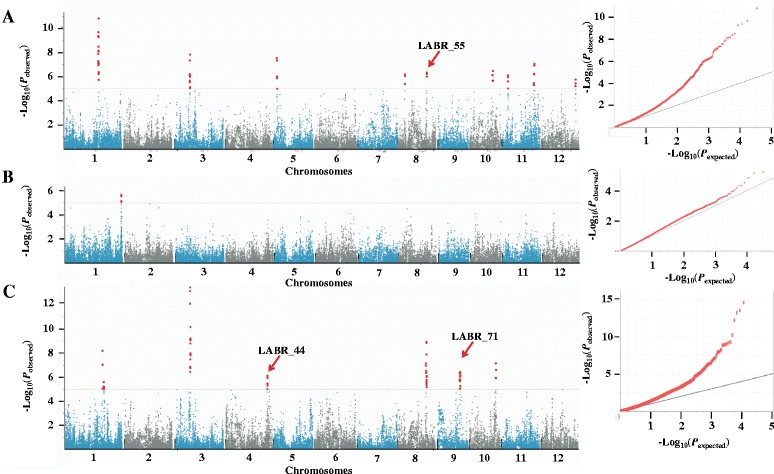
Genome wide association scan for LAFBRs using RDP1 in the three locations. **a** GWAS result in Shanghang, left part, the Manhattan plots of LAFBRs on 12 rice chromosomes, the red arrows indicate that the LAFBRs are co-localized with previously identified LABR regions (LAFBR_7 co-localized with LABR_44, LAFBR_9 with LABR_55 and LAFBR_11 with LABR_71); right part, corresponding Q–Q plot. **b** GWAS result in Taojiang. **c** GWAS result in Wuchang

Using the 44-K SNP data set and the disease resistance scores of the cultivars, we identified 16 non-redundant LAFBRs (Table 2). Among these loci, eight (LAFBR_1, 2, 8, 9, 12, 14, 15, and 16) were associated with the resistance to the *M. oryzae* population in Shanghang, five (LAFBR_3, 4, 7, 11, and 12) were associated with the resistance to the *M. oryzae* population in Wuchang, and only one (LAFBR_5) was associated with the resistance to the *M. oryzae* population in Taojiang. Two loci (LAFBR_6 and 10) were associated with the resistance to the *M. oryzae* population in both Wuchang and Shanghang (Fig. 3a–c). The 16 LAFBRs are located on chromosome 1, 3, 4, 5, 8, 9, 11, and 12 in the rice genome.

**Table 2:** The LAFBRs that are associated with the field resistance in the three field blast nurseries

LAFBRs	Chr.	Positions	Top SNP	Top SNP genotypes	R associated SNP	MAF (%)	Locus reference
LAFBR_1	1	25768623–26002852	id1015310	C/T	T	32.51	
LAFBR_2	1	26034806–26256424	id1015389	G/T	T	31.29	
LAFBR_3	1	28473508–28602492	id1016715	A/T	A	29.90	
LAFBR_4	1	29430517–29612599	id1017391	A/G	G	31.84	
LAFBR_5	1	32130307–32194257	id1027545	G/T	T	14.47	
LAFBR_6	3	11173192–11364668	id3005883	G/T	G	27.91	
LAFBR_7	4	31260971–31367925	id4010692	C/G	C	30.83	LABR_44
LAFBR_8	5	2450054–2703130	id5001423	A/C	C	27.43	
LAFBR_9	8	5278331–5384087	id8001749	A/G	G	30.83	LABR_55
LAFBR_10	8	21688770–21871571	id8006180	A/G	G	25.93	
LAFBR_11	9	16850040–17094483	id9005401	G/T	T	33.01	LABR_71
LAFBR_12	10	16875502–17129435	id10004848	C/G	G	24.47	
LAFBR_13	10	19737153–19958786	id10006307	A/C	C	23.50	
LAFBR_14	11	4415292–4486716	id11001788	A/G	G	29.78	
LAFBR_15	11	23768147–23957505	id11009444	C/G	C	19.55	
LAFBR_16	12	24903601–25001217	id12009011	C/T	C	28.20

**Fig. 4:**
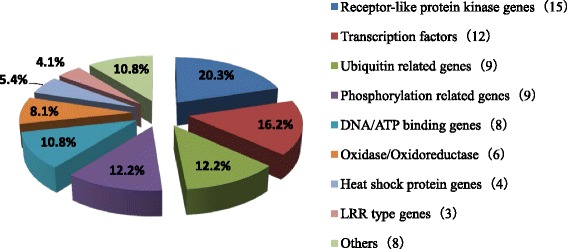
Classification of the 74 candidate genes in the 16 LAFBRs. We manually classified the candidate genes in the 16 LAFBRs using both the MSU (v7.0) rice gene annotation information

Next, we identified 74 candidate genes in the LAFBR regions and obtained their annotation information (Additional file 3: Table S3). None of them are homologous to any known NBS-type R gene family in the rice genome (Liu et al. 2015; Liebranda et al. 2013; Hu et al. 2005) (Additional file 3: Table S3). We classified those candidate genes into nine gene families based on the gene annotation information: 1) 20.3 % (15 of 74) belong to the receptor-like protein kinase gene family; 2) 16.2 % (12 of 74) are transcription factor genes; 3) 12.2 % (9 of 74) are ubiquitin-related genes; 4) 12.2 % (9 of 74) are phosphorylation-related genes; 5) 10.8 % (8 of 74) are DNA/ATP-binding genes; 6) 8.1 % (6 of 74) are oxidase/oxidoreductase genes; 7) 5.4 % (4 of 74) are heat shock protein genes; 8) 4.1 % (3 of 74) are LRR type genes; and 9) 10.8 % (8 of 74) are other defence related genes (Fig. 4).

To measure the expression of the candidate genes during rice-blast compatible and incompatible interactions, we analyzed the RNA-seq transcriptome data sets (Kawahara et al. 2012). When compared with the rice-blast compatible interaction, among the candidate genes, 9.5 % (7 of 74) were up-regulated and 6.8 % (5 of 74) were down-regulated during the incompatible interaction (Additional file 4: Table S4). These 12 genes, whose expression patterns differed between the compatible and incompatible rice-blast interactions, are the strong candidate genes that are associated with field blast resistance.

### Comparison of Phenotypic and GWAS Results Obtained in the Field Blast Nurseries vs. Growth Chambers

To understand rice blast resistance under natural and artificial conditions, we compared the blast evaluations and GWAS results obtained in the three fields in this study with those obtained in the growth chambers in a previous study (Kang et al. 2016). Based on all of the RDP1 cultivars, pair-wise correlation analysis suggested that the resistance level in the field was positively correlated with the resistance level in the growth chamber. The correlation, however, was quite low (*r* values of 0.06 and 0.32) in two cases. The correlation between Wuchang field data and RB22 single-isolate inoculation in a growth chamber is the highest (0.32, *P* < 0.05) (Additional file 5: Table S5). These results suggest that, although the resistance of a few cultivars is similar, the resistance of most cultivars is different in the growth chamber and in the field.

We then compared the 16 LAFBRs obtained in this study with the 97 LABRs previously identified using the RDP1 (Kang et al. 2016) and found that only three LAFBRs are co-localized with LABRs: LAFBR_7 and LABR_44; LAFBR_9 and LABR_55; and LAFBR_11 and LABR_71. Interestingly, none of the 16 LAFBRs was co-localized with R genes previously identified using a traditional mapping strategy. We also compared the 16 loci with previously reported ~70 major rice blast resistance loci including 21 cloned genes (Liu et al., 2014), and didn’t find any overlap regions. Therefore, 13 of the LAFBRs identified in this study are considered to be novel.

### Genotype Analysis of 20 Highly Resistant Rice Cultivars

We identified 20 rice cultivars that exhibited high levels of resistance both in the field (the current study) and in growth chambers (Kang et al. 2016). These cultivars come from 15 countries and belong to different sub-populations (Table 1), including two cultivars in IND, 13 TRJ, one TEJ, one ARO, two ADM, and one AUS. Interestingly, 65 % (13 of 20) of the highly resistant cultivars are from the TRJ sub-population. To determine the genotype of these rice cultivars, we analyzed the haplotype of the 16 LAFBRs in the cultivars (Additional file 6: Table S6). The analysis showed that the average frequency of the R-type alleles for all 20 cultivars is 89.9 %. Among the 20 cultivars, six (301108, 301186, 301229, 301299, 301330, and 301378) contain 100.0 % R-type alleles. These results demonstrate that the R genotypes are highly enriched in the 20 resistant cultivars and that these cultivars could be valuable resistant-donor materials for rice blast breeding as well as for rice blast gene mapping and cloning.

## Discussion

Understanding the molecular basis of field resistance to rice blast is challenging because of the lack of appropriate mapping populations and of a reliable disease evaluation method in the field. In this study, we evaluated the resistance of the RDP1 cultivars in three blast nurseries located in different geographic/climatic rice production areas in China. We found that 61.6, 46.9, and 27.8 % of the RDP1 cultivars are resistant in Wuchang (in Heilongjiang Province in Northeast China), Shanghang (in Fujian Province in Southeast China), and Taojiang (in Hunan Province in Central China), respectively. Through GWAS, we identified 16 LAFBRs involved in field blast resistance. Among these LAFBRs, 13 are novel and the other three are co-localized with known blast resistance regions. One, five, and eight LAFBRs were associated with the field resistance in Taojiang, Shanghang, and Wuchang, respectively, and two loci (LAFBR_6 and LAFBR_10) were associated with the field blast resistance in both Wuchang and Shanghang. This is the first effort to use GWAS in order to identify loci that confer blast resistance in the field.

NBS-LRR type genes form the largest R gene families in plants (Dangl and Jones 2001; Meyers et al. 2003; Jones and Dangl 2006). In a previous GWAS that evaluated single isolates in growth chambers, researcher found that a large number of NBS-LRR type R are associated with the resistance to *M. oryzae* (Kang et al. 2016). In the current study, however, we failed to find an NBS-LRR-type gene among the 16 LAFBRs. The main genes found are those encoding receptor-like protein kinases and transcription factors. These results suggest that the NBS-LRR genes identified in the growth chamber may be more effective against single blast strains than against multiple blast strains. Under blast nurseries conditions, in contrast, the specific NBS-LRR R genes are difficult to detect because of the diversity of *M. oryzae* strains. Consequently, the genes involved in the recognition and signaling of the PAMP-triggered immunity, such as receptor-like protein kinase genes and transcription factors are more likely to be activated in the blast nurseries than in the growth chamber, and these genes may play more important roles in blast resistance in the blast nurseries than in the growth chamber (single isolate condition).

A previous study showed that the most susceptible sub-population (mean disease score = 7.0) in the RDP1 against *M. oryzae* strains in the USA is TEJ (Ali et al. 2010). Similarly, we found that the TEJ group is the most susceptible sub-population to field blast populations in China, and Kang et al. (2016) found that the TEJ group is the most susceptible sub-population to single rice blast isolates. Ali et al. (2010) found that IND is the most resistant sub-population (mean disease score = 3.0) against USA strains of *M. oryzae*. However, our blast evaluations under both artificial and field conditions indicated that TRJ is the most resistant sub-population. These results suggest that cultivars in the TRJ sub-population are a valuable resource for the breeding of rice blast resistance.

We found 40 cultivars that are highly resistant in all three of the tested fields in China. Among the 40 cultivars, 20 are also reported to be resistant against all five of the diverse *M. oyzae* isolates under growth chamber conditions (Kang et al. 2016). Among the 20 cultivars, six (301108, 301186, 301229, 301299, 301330, and 301378) carry 100.0 % of the R-type alleles in the 16 LAFBRs. When checking the background of these 20 resistant cultivars, we found that the following four were reported to be highly resistant: LAC23 (Yu et al. 1996), IRAT13 (Abamu et al. 1998; Chen et al. 2000), Saber (Campos-Soriano et al. 2013), and C101A51 (Chen et al. 2008; Chen 1996; Mithrasena et al. 2012). These cultivars could be used for the breeding of blast resistance in rice and as genetic materials for gene mapping and cloning.

## Conclusions

Using GWAS, we identified 16 LAFBRs associated with rice blast resistance in the field. Among them, 13 are novel and the other three are co-localized in known blast R gene regions. The candidate genes in the LAFBRs encode receptor-like protein kinases, transcription factors, and other defense-related proteins. These results suggest that the genetic architecture of resistance against the multiple strains that are typical at field sites differs from that against the single isolate that are typically tested in the greenhouse or growth chamber. Based the rice transcriptome data, we found that seven candidate genes in the 16 LAFBR regions are up-regulated and that five genes are down-regulated in the incompatible interaction.

We also identified 20 rice cultivars in the RDP1 that confer high levels of resistance to *M. oryzae* under both field and growth chamber conditions. These 20 cultivars will be useful for the breeding of blast resistance in rice. We also demonstrated that the TRJ sub-population is the most resistant group in the RDP1 and is a potentially useful resource for the breeding of blast resistance.

## Methods

### Plant Materials

The rice RDP1, which contains 413 *O. sativa* accessions from 82 countries, was provided by the Genetic Stocks-Oryza (GSOR) Collection, USDA ARS Dale Bumpers National Rice Research Center. The RDP1 represents six major sub-populations, including *Indica* (IND, 87 accessions), *Aus* (AUS, 57 accessions), *Tropical japonica* (TRJ, 97 accessions), *Temperate japonica* (TEJ, 96 accessions), and *Aromatic* (ARO, 14 accessions) (Zhao et al. 2011). The other 62 accessions have an admixed ancestry, and most are classified as *admixtures* (ADM) between *Temperate* and *Tropical* japonica groups.

### Inoculation and Evaluation of Blast Resistance

The RPD1 cultivars were screened in three hot spots of rice blast in the rice production areas of China: Shanghang (in Southeast China, Fujian Province), Wuchang (in Northeast China, Heilongjiang Province), and Taojiang (in Central China, Hunan Province). The 413 tested rice lines were sown and transplanted in the disease nursery beds with 30 plants/plot; the highly susceptible cultivar Lijiangxintuanheigu (LTH) was sown on the plot borders. A randomized block design with two replications was used for the field screens in the three locations.

The RDP1 cultivars were scored using a 0–9 scale for disease severity according to the Standard Evaluation System of IRRI (1996) (Chaudhary 1996): 0 indicates no infection, i.e., immunity (IM); 1 indicates a highly resistant (HR) reaction, with only very small spots on leaves; 2 indicates a resistant (R) reaction, with small lesions; 3 indicates a moderately resistant (MR) reaction, with small elliptical lesions; 4 indicates a moderately susceptible (MS) reaction, with <2 % expanding, elliptical lesion area; 5 indicates a susceptible (S) reaction, with a lesion area <10 %; 6 and 7 indicate susceptible reactions, with a lesion area of 10–25 % and 26–50 %, respectively; 8 and 9 indicate highly susceptible (HS) reactions, with a lesion area of 51–75 % and >75 %, respectively.

### Construction of the Phylogenetic Tree of the RDP1

We re-constructed the phylogenetic tree of the RDP1 using 3835 high quality SNP markers selected from the 44-K SNP markers.

### GWAS Analysis of the RDP1 Resistance in the Field

The methods used to identify LAFBRs were similar to those previously described (Kang et al. 2016). Tassel 3.0 software and the MLM (mixed linear model) were used in the analysis. We re-estimated the appropriate *K* value following the previously published method (Evanno et al., 2015) and found that *K* = 6 is the best for the RDP1 population. Base on the studies on the rice linkage disequilibrium decay at the *Xa5* locus (100 kb) (Garris et al. 2003) and in different *Oryza* species (40–500 kb) (Mather et al. 2007), we selected the associated regions using the following standard: ≤ 250gwkb with at least three significant SNPs (*p*-value ≤ 1E-5).

### Bioinformatics Analysis of the LAFBRs in the Rice Genome

We obtained the DNA sequences of the LAFBR regions from the reference genome of MSU.V7.0 (http://rice.plantbiology.msu.edu/) and analyzed these sequences using BLAT by aligning all the 56,591 rice gene sequences to the LAFBR regions to obtain candidate genes. We classified the identified genes using a similar method previously described (Kang et al. 2016). First, the genes belonging to known R gene families were selected as candidate genes. Second, we expanded our search to defense-related genes that encode the following proteins: NBS-LRR, LRR-TMD, kinase, LRR-kinase or CC-TMD, transcription factor, ubiquitin-related E3 ligase, oxidase/oxidoreductase, protein phosphatase and heat shock protein and etc.

### RNA-seq Data Used in This Study

The RNA-seq data sets were downloaded from the rice-rice blast interaction sequencing project (GenBank accession number: DRX001418 for compatible rice-rice blast interaction transcriptome and DRX001419 for rice-rice blast incompatible interaction transcriptome).

## Additional Files

Additional file 1: Table S1. Rice blast evaluation of the RDP1 in three blast nurseries in the field. (XLSX 39 kb) Additional file 2: Table S2 Forty rice cultivars that were resistant in all three blast nurseries in the field. (XLSX 14 kb) Additional file 3: Table S3. Information on the 74 candidate blast resistant genes in LAFBRs. (XLSX 90 kb) Additional file 4: Table S4. The 12 strong candidate genes in the LAFBRs that are up-regulated or down-regulated in the rice-rice blast incompatible interaction. (XLSX 10 kb) Additional file 5: Table S5. Pair-wise correlation analysis of RDP1 disease reactions evaluated under in the field vs. in the growth chamber. (XLSX 10 kb) Additional file 6: Table S6. The SNP haplotypes of the 20 highly resistant cultivars in the 16 LAFBRs. (XLSX 22 kb)
